# Why the Environmental Data & Governance Initiative is archiving public environmental data

**DOI:** 10.1016/j.patter.2024.101151

**Published:** 2025-01-10

**Authors:** Eric Nost, Gretchen Gehrke, Lourdes Vera, Steve Hansen

**Affiliations:** 1 Department of Geography, Environment and Geomatics, University of Guelph, Guelph, ON N1G 2M7, Canada; 2 Environmental Data & Governance Initiative, USA; 3 Departments of Sociology & Criminology and Environment & Sustainability, University at Buffalo, Buffalo, NY 14260, USA

## Abstract

Public data help researchers and civic organizations develop solutions and advance accountability around environmental challenges but are vulnerable to political threats. While the Environmental Data & Governance Initiative archives data to ensure their availability, we also situate data within their political and economic contexts to support good scientific practice and democratic knowledge production for environmental justice.

## Main text

### The environmental right to know

Governments worldwide collect and distribute data relevant to environmental challenges. For instance, the United States (US) federal government collects satellite imagery, climatological and weather records, and measurements of ambient chemical concentrations. Researchers routinely draw on these data to develop predictive models of conditions and potential interventions, while other groups leverage these data to advocate around specific issues that matter to them, like climate change.

In particular, governments often release records about toxic chemicals—who discharges how much of what and where—following environmental “right to know” principles. These emerged in the 1970s and 1980s as environmental movements sought information about companies’ production methods.^1^ The US Emergency Planning and Community Right-to-Know Act, Canada’s Environmental Protection Act, the European Union’s Public Access to Environmental Information directive, and the United Nations Aarhus Convention are examples of environmental right-to-know laws and treaties; these authorize data programs such as the Toxic Release Inventory (TRI) and the National Pollutant Release Inventory (NPRI). Other data compilations, such as the US Environmental Protection Agency (EPA)’s Integrated Risk Information System (IRIS), the White House Council on Environmental Quality’s Climate and Economic Justice Screening (CEJST) tool, and information on federal websites, do not have as firm of a legal footing. However, these sorts of tools are what public agencies have innovated to help broad user bases understand government data, and they are often the highly valued resources that advocates, practitioners, and policymakers use the most.

The Environmental Data & Governance Initiative (EDGI) is a research collaborative focused on advancing the environmental right to know, especially by developing civic technologies and critical analyses that safeguard and expand public access to US federal environmental data and information. In advance of the second Trump administration, EDGI is preparing to preserve vulnerable datasets and online resources.

### Threats to data

Decisions about which data to collect and whether or how to make them available are inherently political. For example, the government of Alberta, Canada, is proposing to prevent the oil and gas industry from submitting emissions records to the federal government in order to stymie its forthcoming emissions cap. In the US, conservative organizations such as the Heritage Foundation and the America First Policy Institute, whose members comprise key roles in Donald Trump’s second administration, have proposed several policies and actions that would undermine environmental data programs, including eliminating EPA’s IRIS chemical toxicity database, narrowing the scope of the EPA’s Greenhouse Gas Reporting Program, and changing race, ethnicity, and citizenship elements of the US Census.

EDGI has been tracking how these policy proposals build on the first Trump administration’s deregulatory policies and obfuscation of information. For example, we employed web crawling and scraping to determine that, 5 months before the Trump EPA proposed repealing the 2015 Clean Power Plan, it had permanently deleted all webpages, fact sheets, and emissions and financial incentives calculators related to the Plan (see Figure 2 in Nost et al.).^2^^,^^3^ This is one of the various instances where the Trump administration repeatedly suppressed information about programs and issues unaligned with its agenda.

While EDGI did not observe widespread data destruction during the first Trump administration, changes to the legal landscape and an authoritarian style suggest that Trump’s second presidency promises to be different. Federal public data are again vulnerable in this administration, which is not so much anti-science as it is a proponent of “deregulatory science.”^4^ We don’t expect the Trump administration to abandon scientific principles and data wholesale but to weaponize them in service of special economic interests, following a playbook laid out in the 2018 Strengthening Transparency in Regulatory Science rule.^5^ Even if there are data collection requirements that the administration can’t avoid from a legal perspective, there are other ways it can damage datasets. These include reductions in funding or attrition of staff and scientific capacity for programs generating, collecting, and stewarding data^6^; requiring data to be open access even when inappropriate^5^; minimizing access to datasets^3^; or modifying or removing the unprotected web pages where the context (including metadata) necessary to understand the data reside.^2^

The US federal data landscape is already precarious due to its design. It is massive but also extremely siloed—by agency, office, and program—and riddled with incomplete and missing data, particularly where the federal government has not enforced reporting requirements for state agencies and regulated industries.^7^ Each of these issues makes it more challenging for civil society to use public data, let alone safeguard it for future use. Moreover, the federal data landscape is shaped by the technocratic regulatory state, meaning it often does not collect the data needed for addressing issues that actually matter to people (e.g., fenceline and community air quality monitoring).

This is the paradox of public data; it is inherently flawed, but it is often the best evidence available, especially when analyses need to speak to decision-makers at the agencies who provision it. Therefore, utilizing—and archiving—public data requires careful context.

### Importance of data preservation

Preserving public data in accessible repositories is important for science, democracy, and justice. Archiving is good *scientific* practice because it enables continued access for communities of expert researchers to verify, use, and improve the data. Using FAIR principles (findable, accessible, interoperable, and re-usable), archiving supports scientific advancement through productive, rigorous criticism. Archiving is also a good *democratic* practice insofar as it ensures that publics beyond professional scientists can acquire, interpret, and weigh evidence for themselves. Data can also support more *just* decision-making by elucidating who reaps benefits and who bears the burdens of various state-permitted activities, such as facility emissions. These principles underlie the right-to-know laws described above.

However, EDGI has found that archiving data in and of itself will not necessarily lead to scientific advancement or social and environmental progress. Rather, to make archiving data meaningful, we must also account for its context. EDGI has launched an Environmental Enforcement Watch where we’ve not only archived the US EPA’s Enforcement and Compliance History Online (ECHO) database but made it more directly available to partners in user-friendly ways. Doing so has had the added benefit of illustrating some of the limitations of public data systems. In analyzing our archive of ECHO, we found that facilities in majority-minority neighborhoods tend to have fewer records of inspections made under the Clean Water Act.^7^ If we treat data as a commons, where civil society groups are encouraged to make copies of and engage with the data on their own terms, we not only make data more available but facilitate practices of identifying missing data and, from there, can develop recommendations for expanded data collection.

### Our approach

Based on our experiences, we believe that data archiving efforts should approach data as relational and situated in its social and political context. By relational, we mean thinking of data less as a thing in and of itself but more as a set of relationships between phenomena, observations and records, metadata, infrastructures, and practitioners. Data take on meaning not by themselves but within these relationships. By situated, we mean acknowledging environmental data as produced by people and institutions from particular social standpoints and reflecting the interests, advantages, and limitations of those perspectives.^8^ If data archiving is to benefit science, democracy, and justice, it must go beyond supporting raw accessibility to enabling carefully contextualized use.

Ahead of the second Trump presidency, we are working with partners to identify specific vulnerable and irreplaceable federal resources rather than attempting to create copies of every single dataset. Then, EDGI and partner organizations are moving to archive, curate, and distribute these resources, including datasets and metadata with context on their origins and purpose. This involves overcoming challenges in automating crawls and scrapes of interactive web pages, which are often how federal agencies distribute data (rather than through bulk downloads or APIs).

EDGI’s archiving efforts are focusing on datasets that reflect communities most vulnerable to Trump’s anticipated policies, such as the proposed dismantlement of EPA environmental justice programs and undermining of race/ethnicity components on the US Census. Data have become a target of conservative agendas because undermining climate action and countering gains made by justice-seeking groups, in order to further fossil fuel and other industry interests, involves diminishing the collection of relevant data. To this end, we will archive CEJST, EJScreen, and ECHO, among other datasets, because they document environmental injustices and their impacts. In this, we are guided by the CARE principles for Indigenous data sovereignty, which pay attention to the potentially good or harmful relations between stewards, users, and those data impacts.^9^

We strive to archive whole data *projects*, including documentation and additional context, as well as datasets. While the technical standards for developing metadata and data provenance are important, we need to document the fuller stories of datasets’ origins. For instance, making full use of datasets like TRI, NPRI, or ECHO requires understanding the permission to pollute system whereby the US and Canada permit companies to release toxics under thresholds of “safe” amounts established by regulatory agencies in relation to political and economic contexts that shape how these thresholds and pollutants are even perceived.^10^ High-profile studies have used ECHO data, for instance, without situating this context, reporting violations of environmental protection laws as if the records speak for themselves and without addressing what constitutes a violation or how the origin and purpose of the data influence that. Archives of both the “raw” datasets and those generated from analyzing them would need to highlight this for users, perhaps in the form of model cards or datasheets.^11^^,^^12^

### Conclusions

Archiving public environmental data is important for scientific research, democratic participation, and environmental justice. But missing, siloed, and biased data that don’t match communities’ needs means we must both reconsider the assumption that more data automatically lead to better outcomes and account for its political and economic context. We propose a collaborative approach to archiving, involving partnerships with diverse organizations to identify, preserve, and contextualize public datasets. A more critical and responsible approach to data archiving goes beyond mere accessibility to ensure that data can be used in a way that promotes equity and justice.

## Declaration of interests

The authors declare no competing interests.
